# A review on the structural characterization of nanomaterials for nano-QSAR models

**DOI:** 10.3762/bjnano.15.71

**Published:** 2024-07-11

**Authors:** Salvador Moncho, Eva Serrano-Candelas, Jesús Vicente de Julián-Ortiz, Rafael Gozalbes

**Affiliations:** 1 ProtoQSAR S.L., CEEI Valencia, Avda. Benjamin Franklin 12, 46980 Paterna, Spain; 2 Universitat de València, Facultad de Farmacia, Departamento de Química Física, Unidad de Investigación de Diseño de Fármacos y Conectividad Molecular, Avda. Vicent Andrés Estellés 0, 46100 Burjassot, Spain; 3 MolDrug AI Systems S.L., Olimpia Arozena Torres 45, 46108 Valencia, Spain

**Keywords:** descriptors, nanomaterials, nano-QSAR, QSAR, toxicity

## Abstract

Quantitative structure–activity relationship (QSAR) models are routinely used to predict the properties and biological activity of chemicals to direct synthetic advances, perform massive screenings, and even to register new substances according to international regulations. Currently, nanoscale QSAR (nano-QSAR) models, adapting this methodology to predict the intrinsic features of nanomaterials (NMs) and quantitatively assess their risks, are blooming. One of the challenges is the characterization of the NMs. This cannot be done with a simple SMILES representation, as for organic molecules, because their chemical structure is complex, including several layers and many inorganic materials, and their size and geometry are key features. In this review, we survey the literature for existing predictive models for NMs and discuss the variety of calculated and experimental features used to define and describe NMs. In the light of this research, we propose a classification of the descriptors including those that directly describe a component of the nanoform (core, surface, or structure) and also experimental features (related to the nanomaterial’s behavior, preparation, or test conditions) that indirectly reflect its structure.

## Introduction

Computational techniques of statistical nature such as quantitative structure–activity relationships (QSARs) can help to understand the intrinsic features of nanomaterials (NMs) and quantitatively assess their potential risks for human health and the environment [1]. QSARs consist in the construction of mathematical models relating the structure of a series of molecules to a biological/physicochemical property or activity, mostly through the use of statistical tools. Once a model has been constructed, it can be used to predict the property or biological effect of new structures quickly and at a very low cost in comparison to experimental approaches. Furthermore, when they are developed complying strictly with the rules established by the Organization for Economic Co-operation and Development (OECD) for their scientific validation, QSARs are accepted for regulatory purposes, thus ensuring their applicability at the regulatory level by international bodies such as the European Chemicals Agency (ECHA) [2–3].

Unlike QSAR models for discrete organic molecules, QSARs for NMs are still at an early stage, mainly because of the lack of data available regarding their generation [4], but also because of the intrinsic difficulty to characterize the structure of NMs [5–7]. The first described nano-QSAR model is from 2009 [8], but the number of relevant nano-QSAR models is growing significantly because new nanoscale descriptors are found [6], and more information on NMs is progressively generated, opening new ways of improving nano-QSARs. This is an active field and, recently, a comprehensive review about this topic and the future perspectives was published [7]. Scientific, industrial, and national institutions should harmonize their efforts for the development and application of nano-QSARs at the regulatory level [9].

From a regulatory point of view, ECHA recognizes the complexity of NMs and the fact that the same chemicals could lead to different nanostructured substances, which, despite sharing the chemical composition, should be considered different materials in terms of their activity and properties. ECHA uses the term “nanoform” to specify a particular substance in the NM field for questions such as their registration and risk evaluation. A nanoform is defined by having particles with a specific composition and with structural properties (such as size and shape) in a defined range. In this way, it differs from more general labels used for NMs (e.g., “Au nanoparticles”) to refer to a family of materials combining different sizes and/or coating materials that can have different properties. Hence, ECHA defined a set of relevant physicochemical parameters to identify and register nanoforms, including six compulsory requirements, namely, composition, impurities, surface treatment functionalization, size, shape, and surface area [10].

**Figure 1:**
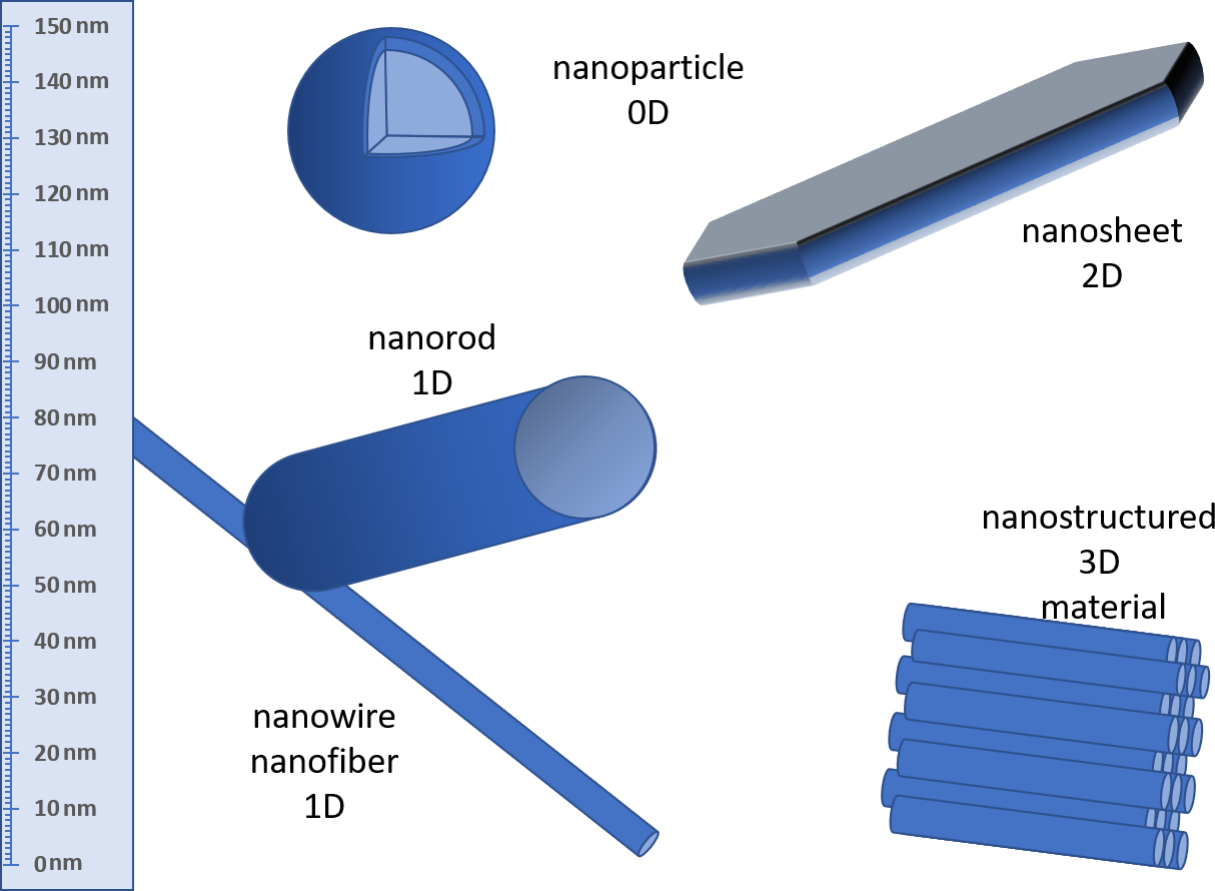
NMs with different kinds of shape. The number of dimensions next to the names refers to those that exceed the nanoscale.

One of the challenges in nano-QSAR modelling, and in the modelling of NMs in general, is the definition and the identification of what a single NM is. Discrete organic molecules can be fully identified and characterized by their chemical structure, often represented by a SMILES code [11]. This approach is insufficient for NMs, as a key component of their definition is their size. NMs are defined as materials with at least one of the dimensions (including internal features) on the nanoscale (1–100 nm). Figure 1 shows some types of NMs according to their dimensions [12].

Several studies show that the nanoscopic structure of the nanoparticles or their aggregates affects the behavior of NMs, and more particularly their toxicity. The influence of the size and the structure of nanoparticles or their aggregates on their toxicity has been recently reviewed [13]. From now, we will use the label “nanostructure” to refer to these properties, in comparison with the term “structure” referring to the chemical composition. The nanostructural differences among nanoparticles can be defined by different means: (i) direct measurements of their structure (e.g., their size), (ii) comparison of their physical properties that depend on size/nanostructure, and (iii) consideration of differences in their preparation.

**Figure 2:**
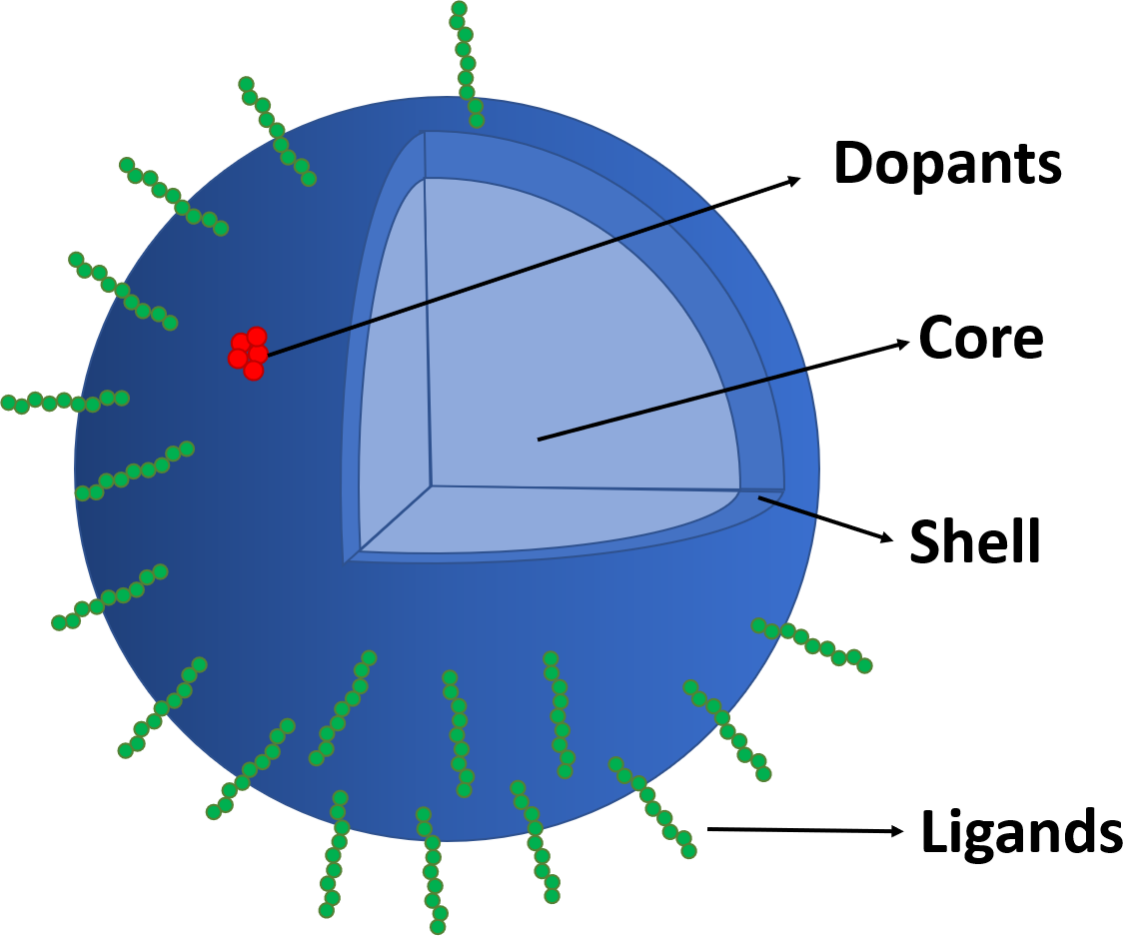
Schematic depiction of the parts of a complex nanoparticle.

Another particularity of NMs is their chemical composition, as they could exhibit complex compositions (Figure 2) formed by different parts such as (i) the core (the inner part of the NM and most of its weight), (ii) the shell (the composition of the surface that interacts with the solvent and biological molecules), (iii) impurities or dopants (minor components deposited on the surface or distributed among the material that affect the properties), and (iv) ligands or coating (organic molecules linked to the external part of the particle that contribute to its formation, solubility, or function).

Moreover, different experimental factors during the life of a NM (i.e., the conditions during its preparation and handling) will lead to different structural configurations and to different properties. Thus, the reported experimental conditions are significant, and they often need to be included in a predictive model.

Finally, the quality of data is a key component in the development of QSAR models. Consistency of the data is a key aspect in the preparation of a database for a QSAR study as different changes in the conditions of the test could lead to a dispersion of the results [4]. This should be considered carefully when collecting data from different sources. It becomes a harder problem in nano-QSAR as not only differences that can arise on the evaluation of the endpoint must be considered, but also those regarding preparation of the NMs and the way they are identified. In addition, the inclusion of experimental values as descriptors further reduces the availability of data on NMs that have been tested for both the adequate characterization results and the prediction endpoint.

Therefore, the development of QSAR models requires the codification of information on nanoforms beyond classical molecular descriptors. NMs have some particularities in comparison with discrete substances, which are (i) the importance of the size and shape, (ii) the complex composition, and (iii) the consequences of the preparation of the NM on their features. All these particularities need to be codified somehow as NM descriptors (nanostructural features) that are the basis of the development of nano-QSAR models (in a similar way that molecular descriptors are fundamental for QSAR models). However, the challenge goes further than describing numerically the structure. These aspects also have to be considered in the recording and identification of the NMs. A recent approach to this issue from Lynch et al. is the development of InChI codes for NMs, which expand the InChI codes used to identify chemicals [14].

In the present work, we have collected and analyzed the existing models in the literature and how different authors address the codification of NMs. Moreover, in an attempt to harmonize NM modelling, we propose a new classification of NM descriptors.

## Review

### Available nano-QSAR models

We have surveyed the literature to compile the existing models and to analyze the variety of calculated and experimental features used to define and describe NMs. A total of 77 different publications including NM-focused prediction models have been found, and the information is collected in Table S1 (Supporting Information File 1). This review is not restricted to self-considered nano-QSAR models, but it includes also other predictive models (such as Bayesian networks or mapping strategies) that use calculated and/or experimental features that could potentially be used as descriptors in a nano-QSAR model. For the literature analysis below, all descriptors with a potential use in nano-QSAR are discussed.

### Descriptors for NMs

**Figure 3:**
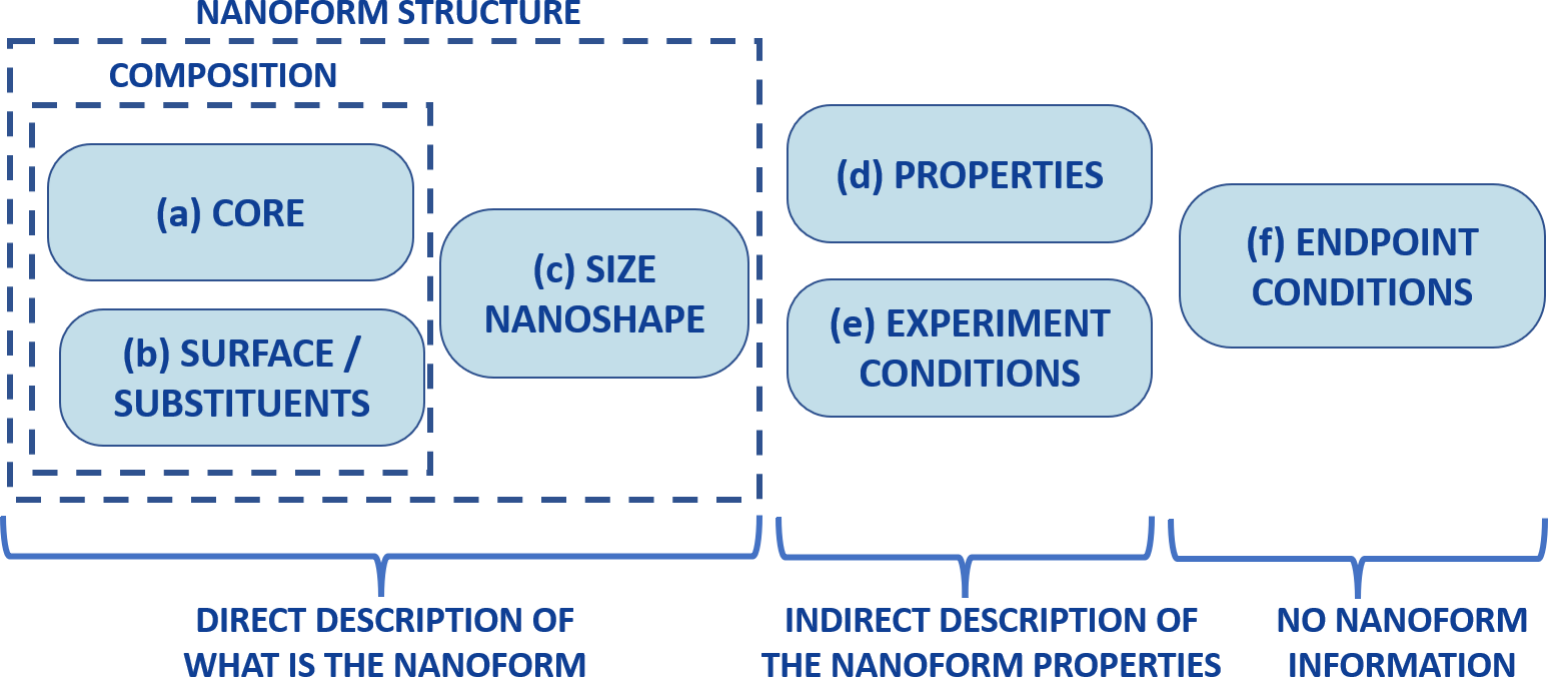
Classification of nano-QSAR descriptors.

One of the conclusions of the analysis of available models is the heterogeneity of the criteria used by different authors to characterize the NMs [7]. Taking these models as starting point, and in order to harmonize the characterization of the nanoforms, we propose a classification of the descriptors as follows (Figure 3): (i) Descriptors that directly describe the nanoform, that is, its chemical composition or its physical structure. Descriptors based on the chemical composition are similar to those used in QSAR models of discrete molecules. Nevertheless, in nano-QSAR, the descriptors should differentiate between those describing the main component of the nanoform (the core, (a) in Figure 3), those related to the external part (shell or surface) and/or the substituents or ligands attached to it ((b) in Figure 3), and those that directly reflect the nanostructure of the nanoform (including factors such as size, aspect ratio, or surface area, (c) in Figure 3). (ii) Descriptors that codify experimental information on the NMs and do not directly describe the composition or structure of the NM, but can be used to model them because they imply nanostructural features and composition. We assign different groups to these experimental measurements, depending on whether they describe properties that are consequence of the structure of the nanoform (e.g., wavelength or zeta potential, (d) in Figure 3) or whether they represent experimental conditions that contribute to the formation of nanoforms and are the cause of their structure (such as the synthesis medium or the time span between preparation and testing, (e) in Figure 3). (iii) Descriptors related to the experimental conditions of the determination of the endpoint. It is possible that some of those conditions also affect the structure of the nanoform in experimental media; however, these descriptors are not focused on the nanoform itself but on the measured endpoint (such as the target or exposure time, (f) in Figure 3.

#### Descriptors that define the nanoform

**Figure 4:**
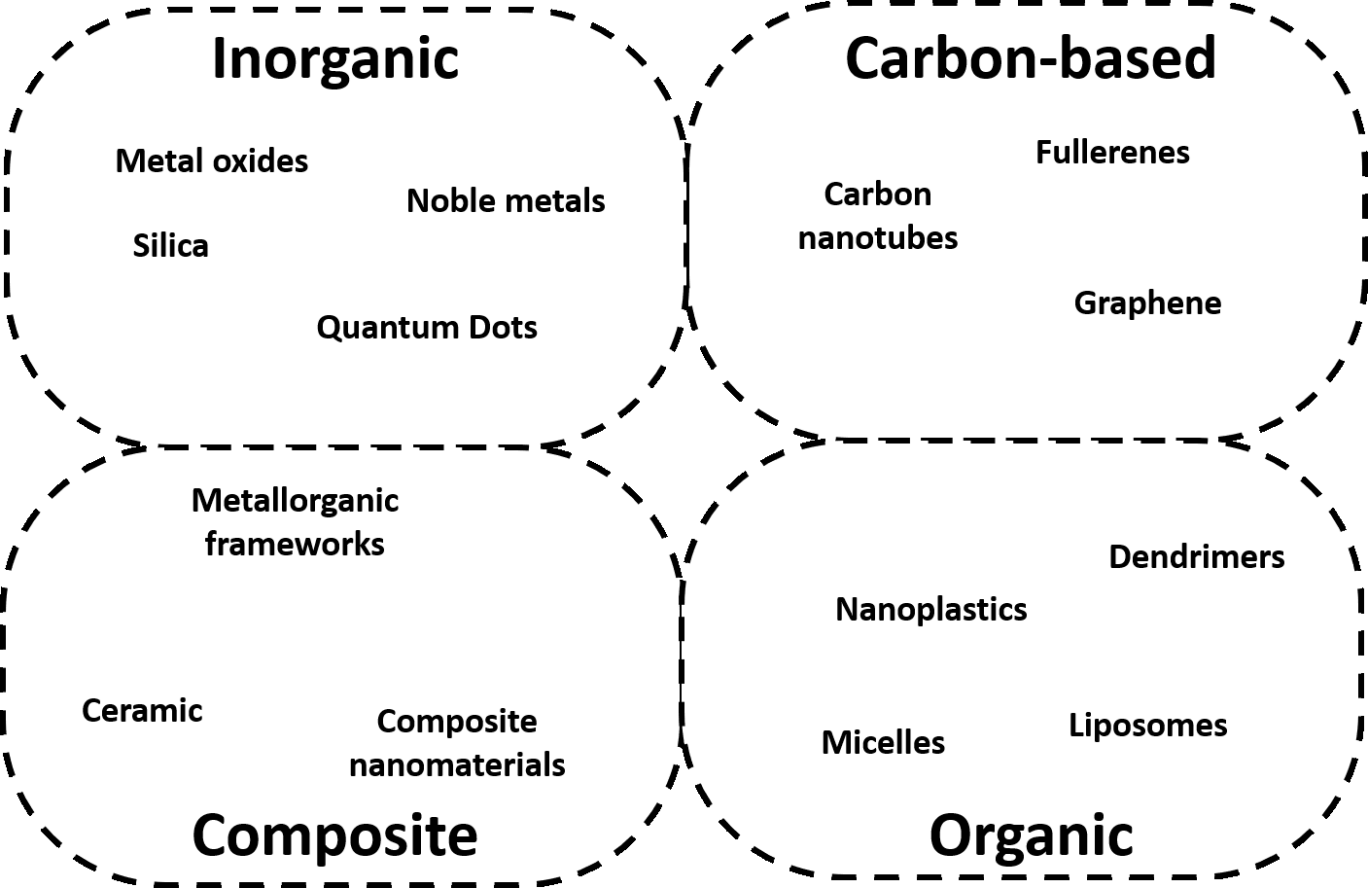
Classes of NMs by chemical composition.

**Core composition (a):** The first family of descriptors are those that describe the core composition of the nanoform. This kind of descriptors can be applied depending on the type of nanomaterials, which can be classified according to their chemical composition in inorganic, carbon-based, organic, and composite NMs (Figure 4). In organic molecules, a wide range of descriptors are obtained from the topology of the molecule, arising from the rich variety of structural motifs that can be found and the relevance of their distribution along the molecule. However, the core of the NMs is typically composed by chemicals with a simpler and repetitive chemical structure. Most inorganic materials are composed of single elements (e.g., Au or Ag) or binary compounds (e.g., Fe_2_O_3_, CdSe, or SiO_2_). The most abundant families among the carbon-based NMs are nanotubes and fullerenes; they are also considered inorganic and have a simple chemical composition (mostly carbon). Hence, classical organic molecular descriptors are not commonly found in the core composition, although they are potentially applicable to structures involving organic polymeric substances (such as nanoplastics and dendrimers) or lipids (such as liposomes).

Because of the simpler chemical structure of the components typically found in NMs, the chemical descriptors tend to be also simpler than those of organic molecules. Furthermore, it is common to find nano-QSAR models focused on groups of nanoforms that have different activity but are chemically homogeneous in their core, that is, which include NMs with the same or similar core composition (e.g., only nanotubes and fullerenes, or only metal oxides). One example is the use of the count of metal and oxygen atoms as descriptors in metal oxide models [15–16].

It is also common to find single-element descriptors based on the physicochemical properties of pure elements. The use of single-element descriptors is trivial in single-metal nanoforms, such as silver or gold nanoparticles [17]. In other cases, a weighted-average can be used to transform the element-based descriptors to the current composition of compounds [18–19]. Most of the descriptors are based on the empirical formula (i.e., the proportion of elements in the substance), such as the molecular weight, which is calculated from a symbolic formula [15,20–21], or descriptors calculated from element-based values transformed to the empirical formula [20]. However, because of the common presence of oxygen, several descriptors of metal oxide models do not take into account the oxygen atoms and depend only on the identity of the metal, such as metal mass [15], electronegativity [16,20,22]_,_ or position in the periodic table (group and period) [15–16].

Despite the fact that, generally, such element-based descriptors are independent of the compound, in some cases they are related to the particular composition of the material, such as oxidation state, formal charge [8,16,20,23], softness [22], ionization potential [22], and weight percentage of the metal [23]. Furthermore, to include information regarding the particular nanoform, the crystal structure can be included as a categorical descriptor [24] or by using coordination numbers [24]. Alternatively, Kotzabasaki et al. also codified the composition of iron oxide nanoparticles with a single categorical descriptor that encodes the crystal structure of the main component (in this case as maghemite or magnetite) [25].

Alternatively, some descriptors are focused on the complexity of inorganic materials and, in place of structural features, focus on electronic features. In this regard, several descriptors were obtained from quantum mechanics (QM) calculations of small clusters or periodic models [26–30]. Although cluster-calculated QM descriptors are inherently size-dependent, they are calculated using smaller, single-size model clusters, which are not related to the size of the nanoparticles; thus, they should be considered size-independent. Cluster-related values include standard heat of formation, total energy, electronic energy, core–core repulsion energy, area and volume of the cluster, energies of HOMO and LUMO orbitals and the gap between them, and lattice energies [22,26]. The energy levels of conduction and valence bands, which are found commonly among the most important parameters, can be calculated from QM models or derived from other simple reference parameters by empirical formulas [31]. Additionally, QM calculations can be performed in very simplified models that only describe a part of the material, such as single metal atom, to calculate the enthalpy of formation of the cation [26]. However, there is an alternative, simplified way of incorporating the electronic structure in the model, that is, by using the electron configuration of the elements (e.g., by using electron configuration fingerprints) [32]. In this way, the atomic orbitals can be easily represented and used to estimate the molecular/crystal orbitals in the NM without requiring an electronic calculation.

Also, experimental physical properties of the compound, obtained from classical databases or literature sources, could be used. However, because these measures correspond to the bulk material and do not characterize the nanoform, we classify them as composition-related descriptors and not as experimentally measured physical descriptors of the investigated nanoform. Examples of this are the atomization energy of the bulk MO*_x_* structure obtained from literature sources, used by Liu and coworkers [15], and the formation energies used by Banerjee and coworkers [33]. Nano-QSAR models based on the CORAL software [34] use descriptors that are optimized from the dataset by using an identifier text string called “quasi-SMILES”, an extension of the SMILES incorporating in a single string the composition of the core and additional information related to the nanostructure or the conditions [35–37]. However, both data are not always combined; the first model by Toropov et al. restricts its textual descriptor to a simple SMILES representation of the molecular formula of the metal oxide [38], and there are quasi-SMILES descriptors without any composition data [39]. Nevertheless, the nanoform identification of certain materials, such as pristine carbon nanoforms, does not really describe the composition (pure carbon) but the nanostructure (shape and composition of nanotubes or fullerenes) [40].

Otherwise, the single-formula representation of the chemical composition of a nanoparticle discussed until now can be simplistic. NMs are often found to include different chemical components because they are mixtures or complex chemical structures, including impurities or even different crystal phases. Hence, the primary chemical composition of the nanoparticle, excluding ligands or external substituents, can be categorized into two parts, the core and the shell compositions. This approach was employed in a study of quantum dots [30], which does not use numerical descriptors for the composition, but directly uses a Bayesian network with categorical descriptions of both the core and the shell. These are determined by empirical formulas of either a single inorganic salt or a mixture. Additionally, a distinct category has been designated for quantum dots lacking a specific shell composition, labeled as “non-coated” [41].

**Substituents and modifiers (b):** The consideration of substituents or modifiers on the surface of a NM is essential to identify and describe the nanoform as they may influence its properties. The characterization of these substituents or modifiers becomes even more relevant when the core composition among different NMs is the same (e.g., silver), but the substituents differ (e.g., different organic ligands). The substituents can be organic chemicals as, for example, in the model developed from the database by Weissleder et al. [42] for several same-core superparamagnetic nanoparticles functionalized with different organic molecules. The authors used SMILES-based descriptors, common in QSAR models of discrete molecules, to characterize the substituents, and they constitute the only identifier of each datapoint [43–46].

In datasets with substituents that are mainly transition metals (deposited in the nanoparticle or present in the solution), properties such as the ionization potential, the electron affinity, the absolute electronegativity and the absolute hardness, as well as the adsorption energy of the metal have been used (using literature values or QM calculations) [47–50]. In those cases, the descriptors were obtained for a single component, and the final value of the mixture was calculated as a linear combination weighted by the molar fraction. In other cases, the molar composition of the metallic substituent was also directly used as descriptor [51]. The idea of considering a NM as a mixture and developing QSAR models for the toxicity of nanoscale mixtures formed by a NM and discrete molecules or ions, which could potentially work by attachment to the surface, has been reviewed by Trinh and Kim [52].

Similarly, carbon-based nanoforms are constituted of a common carbon core, but they can have different side groups attached to the surface. In the case of C_60_ fullerene structures (with the same exact fullerene composition), the datapoints were identified merely based on these side groups only. The corresponding molecular descriptors comprised 3D QM-calculated descriptors (which include the constant fullerene) and descriptors only based on the structure of the functionalization group [53–54]. Coating descriptors are not only found in common-core models. For example, Kleandrova et al. included descriptors based in the bond adjacency matrix for the organic coating if present (a zero was used for uncoated nanoforms) [18].

Bilal et al. did not use numerical descriptors to describe the composition, but categorical descriptors that included empirical formulas for the core and the shell, as well as different categories (one group and one specific name) for each of the ligands and surface modifications in a Bayesian network [41]. Similarly, categories for the ligands are used in a quasi-SMILES-related model for single core–shell quantum dots [55]. Alternatively, other authors combined all components in a single fingerprint without differentiating the composition of core and coating [32].

**Size and nanoshape (c):** The most direct and common approach to describe the nanostructure of a NM is to include values that provide a physical description of the particle. Particle size is the most common feature in nano-QSAR models. However, despite the established understanding that size plays a crucial role in the activity and toxicity of NMs, its significance in the performance of QSAR models seems debatable. For example, the original nano-QSAR models of Puzyn et al. [26] and Gazewicz et al. [30] used only core-related descriptors, and they argued that the size does not significantly affect the property investigated for NMs in a predetermined size range (15–90 nm). Thus, even though subsequent studies on similar datasets considered the size of the nanoforms as a descriptor, because of the limited size range in the training database, it is common for size not to be among the most relevant descriptors in the models [56–57]. At the other extreme, there are studies where the only difference among the nanoforms used (without considering endpoint-related descriptors such as dose or time) is the size [58–59].

The size of nanoparticles is commonly measured by transmission electron microscopy (TEM). TEM images can provide several descriptors that reflect the nanoform’s shape and size, such as its area, volume, surface, diameter, volume/mass ratio, volume/surface ratio, aspect ratio, porosity, sphericity, and circularity [30]. However, the most common approach is to provide a single size parameter and assume that the nanoparticles are approximately spherical [23,35,56,60]. In some cases, the length in a second direction is also reported or, more often, a ratio between two dimensions is included to encode the shape of the nanoparticle or to categorize it [60]. Alternative size parameters are volume and mass [61].

Dynamic light scattering (DLS) is another technique that can be used to describe the hydrodynamic size or the aggregation of the nanoforms in larger nanostructures, depending on the medium and other conditions. In some cases, the size values reported in the papers are not measured on purpose, but are the nominal values found in the vendor's documentation. Some authors have reported the TEM diameter as primary size, but included also values for the hydrodynamic diameter measured by DLS [23,62–63], even in some cases in different media such as ultrapure water and a different medium (i.e. buffered [64] or bacterial [56] media).

Additionally, categorical variables describing the kind of structure can be used to reflect the shape. For example, a shape component in mixed carbon-based nanoparticles is encoded by this type of categorical variables, such as fullerene vs carbon nanotubes or different carbon nanotubes [40,65]. Alternatively, Trinh and collaborators directly encoded the size of multiwalled nanotubes by both their diameter and length. They also included the surface area as a structural descriptor, by using a hierarchical clustering method to classify the values in ten categories [66]. The categorization of the size and other physicochemical parameters was required in the quasi-SMILES descriptors used in those cases [51] as they are converted to a string, even when the effect is reduced by dividing the dataset in two categories only with a single size threshold [60]. However, in more modern approaches, quasi-SMILES allow for numerical values for the size and similar experimental values [36].

A different approach to describe the shape and size is provided by the use of the calculated molecular weight for discrete carbon nanoparticles, as well as their calculated surface area (overall and specific) and volume [67]. However, this requires to have the full atomistic description of the nanoform, which is not available for most experimental datasets. Some authors propose additional topological descriptors that are specific for carbon nanoshapes with known topology, such as carbon nanotubes, graphene, and fullerenes. For example, the sum of degrees around the carbon atoms at the surface can be used for all pristine carbon nanoforms [67].

Theoretical calculations of the surface area are more common [68], but it can also be obtained experimentally from gas adsorption data, using the Brunauer–Emmett–Teller (BET) theory [33,51], or directly from the vendor [61]. The surface area can be expressed as total surface area (by nanoparticle), specific surface area (by weight), or both [24].

Finally, the existent size-dependent descriptors should be included in this section. These descriptors are calculated, numerical factors derived from the size of the molecule and other physicochemical properties of its components. For example, a series of size-dependent descriptors, such as the ratio of surface molecules, which involves both the nanoform size and the aggregation size, can be calculated using a liquid drop model approximation [69–70]. This model defines the forces between molecules assuming that they behave like particles in a liquid drop. It uses the estimated Wigner–Seitz radius to calculate the average distance between particles, used as descriptor by some authors [71]. Similarly, the size-dependent electron configuration fingerprints describe mainly electron population, but they also consider the size of the NM and the distribution of the different components to yield an overall single fingerprint of the NM [32].

A different approach is to include the information on the nanostructure not directly as a descriptor, but as a different part of the model framework that contributes to the prediction. For example, the multi-task QSAR model of Ambure et al. [72] mainly uses descriptors based on the core chemical structure, but also a different kind of categorical parameters, labelled as conditions, which modify the descriptors used in the prediction. These are mainly endpoint-related values, but the nanoparticle size is also included as a condition that modifies three of the descriptors using a Box–Jenkins approach [73]. Halder et al. also included the size as one of the perturbation parameters [19]. Other authors included structural features in perturbation QSAR toxicology models, both as one of the descriptors (the size) and as a perturbation criterion (the shape) [18,74–75]. Interestingly, they also included among the perturbation criteria the experimental conditions of the size measurement, which was applied both to the size itself but also to the electronegativity [74–75].

#### Indirect descriptors of the nanoform properties

**Experimental measurements (d):** Because of the complex structures of NMs, it is challenging to understand how the nanostructure affects their chemical and biological activities. However, it is possible to use direct experimental measurements that describe the behavior of the nanoparticle, for example, their electric or chemical properties, in place of their structural features. The rationale behind this usage is that the experiments measure properties that are involved in the activity modelled or that have a structural origin related to the activity mechanism.

A very common property included in several models [23,44,60,68,76] is the zeta potential (a measurement of the charge at the surface of the NMs). The zeta potential value used as a descriptor can be measured in a test medium or in different media, such as water at a specific pH or purity level [15,64,77]. A further step, proposed as an example of combining preexisting structure–activity predictive models in networks, is the prediction of the zeta potential in the relevant medium using a model that uses the measurement in pure water (first layer) and another one that allows for estimating the value of the zeta potential in the ionized medium (second layer) using the output of the first layer [78]. Although the zeta potential is most often included as a numerical value, it can be also used to group the data into categories [60]. Related measures are the isoelectric point, which corresponds to the pH at which a nanoparticle suspension has zero zeta potential [15,17], the surface charge [31,36,63], the conductivity [77], and the electrophoretic mobility [77].

Magnetic properties are also found to be used as NM descriptors, such as the relaxivities *R*_1_ and *R*_2_ obtained from magnetic resonance studies [44]. Related to magnetism, Kotzabasaki et al. used the magnetic field strength, but also a single categorical descriptor describing the magnetic core composition of the nanoparticles [25]. Additionally, focusing on the role of the NM as contrast agent in magnetic resonance imaging, the authors added the specific property of cellular internalization of iron, measured as the amount of iron inside the cells [25].

Zhang et al. [79] created a predictive model that uses regression trees to predict the toxicity of metal oxides using two parameters, namely, the experimentally measured concentration of the metal (expressed as a percentage) and the conduction band energy, which was calculated from different physicochemical constants and also from experimental measurements of zeta potential and diffuse reflectance UV–vis spectra). Alternative formulations for valence and conduction band energies, based only on pre-known physicochemical constants and values from reference handbooks, have been reported as well [79–80].

Furthermore, the electric characteristics of the nanoparticle surface can be reported by its interaction with other substances, as for example using the maximum salt concentration in the medium with no significant coagulation or the rate constant of its oxidation by hydrogen peroxide [68].

It should be noted that the use of experimental descriptors can be exclusive, and there are models such as those of Liu et al. [76] and Fourches et al. [44] that describe a series of NMs with different compositions, including different iron oxides and quantum dots, only on the basis of their size, magnetic values, and zeta potential, without any direct consideration of the composition (i.e., no descriptor of the category “a” or “b” in our classification). Kudrinskiy et al. also modelled silver nanoparticles with different coatings without introducing directly the capping agent in the model, but only by observing the differences in size, reactivity, and electric behavior of the nanoforms with different capping agents [68].

A different approach to the use of experimental properties are models that combine composition-based descriptors with experimental information on the toxicity to different species, such as the interspecies iQSTTR models developed by De et al. [81] and the nano-QTTR development for aquatic toxicity by Jung and coworkers [82].

Finally, we can consider a variation of this type of descriptors, that is, the use of experimental results for specific signaling-pathway responses in order to assess the overall toxicity and to group different NMs together [83–84].

**Experimental conditions (e):** Finally, some descriptors do not directly describe the nanoform; instead they consider how it was prepared. In this context, the effects of the preparation methodology could be assessed without describing the specific structural features that arise from the preparation. In contrast to the following group (f), we have reserved this to experimental conditions of the processes performed prior to the test of the predicted property. These conditions lead to a specific nanoform, even if there is no characterization step to identify its properties, that is used for the test and, potentially, for other independent tests. Hence, differences in equivalent tests should be related to underlying differences in the nanostructure.

The wide range of attributes selected by Liu et al. [85] in their predictive method of toxicity based on a combined index for zebrafish (EZ metric) included the synthesis precursors. Similarly, Gul et al. compiled a dataset of nanoforms in cell viability tests to perform an association rule mining analysis in which the synthesis method was included among the identifiers of the nanoparticles [86].

In another example, in the read-across models developed by Varsou et al. [77], the effect of aging the nanoparticle for two years prior to toxicity testing has been considered. However, instead of including this as a descriptor, they provided values for some of the experimental descriptors measured before and after aging. They also concluded that discriminating aged from pristine nanoparticles improves the predictive value of the model.

#### Descriptors independent on nanoforms

**Experimental endpoint conditions (f):** This section includes descriptors that codify information about the experimental conditions of the test that potentially affect the value of the measured parameter of the endpoint. The exposure of the NMs to different conditions could produce structural changes, which could be responsible for changes in their activity. The NM particles are known to be significantly affected by the medium regarding size, aggregation, ligands, and nanostructure. Nevertheless, although the parameters considered here could have direct impact on the value, their relevance could not be directly linked to structural differences in the nanoform, in contrast to the conditions classified above in group (e).

Such descriptors are commonly found in multi-task QSAR models, where different endpoints are modelled using the same framework. For example, it is possible to have different target cell lines (identified by one or more descriptors) [24,31,36,60,66] or to combine different toxicity assay methods [24,36,63,66] in the same model.

In some models, binary descriptors are used to indicate the absence or presence of a certain condition such as centrifugation, stirring, sonication, dispersion, or presence of additives [17,39,65,87]. Numerical descriptors used to encode the test environments include the ionic strength [17], the amount of organic matter [17], and the pH value. More specific variables can be found for particular tests, such as the number of daphnia individuals in an immobilization test [17]. Also, descriptors that quantify the exposure to the nanoform, such as exposure time [17,31,37,60,66] and dose [31,37,63,66] are very common in nano-QSAR models.

A different approach of multi-task QSAR models to incorporate the endpoint conditions is to use them as modifying factors of the descriptors. For such a modification, using a Box–Jenkins approach, Ambure et al. [72] classified the dataset based on two endpoints and several experimental protocols, cell line targets, exposure times, and doses. Other authors use perturbation QSAR models to incorporate endpoint conditions such as the specific toxicity measurement [18,74], the biological target [18–19,74], the exposure time [18,74], and the incubation conditions [19].

Although not directly used as a descriptor, it is worth to note that Pathakoti et al. [61] included the light exposure as a variable in their toxicity models of metal oxides versus *E. coli*, obtaining two series of toxicity data for the same set of NMs. Analogously, Basant and collaborators considered toxicity values measured under different light conditions in *E. coli* and in HaCaT cells in a multi-target QSTR model [88].

## Conclusion

In this review we have analyzed in depth the descriptors used in the literature in QSAR and related in silico prediction models for NMs. Our review highlights that the high degree of variability in the NM properties is a key challenge in nano-QSAR models, because it makes it difficult to develop models that are accurate and generalizable across different NM types. Thus, most nano-QSAR models are based on data sets limited to very similar nanoforms, which can lead to overfitting and poor predictions out of the applicability domain. Regarding the kind of descriptors used, there is a significant variety of descriptors including low- and high-level calculations, qualitative classifiers, and experimental features.

Furthermore, it is difficult to find common points such as the requirement of a particular set of features for each kind of nanoforms. For example, key features such as the composition of a NM or its size are not included in all the models. It should be noted that some nano-QSAR models have been developed based exclusively on testing conditions (e.g., dose, preincubation, and sonication) of a single nanoform. In these cases, the chemical structure and direct structural information are constant and do not need to be included among the descriptors.

The descriptors found throughout 77 publications have been classified based on the information that they codify (Figure 3). This classification proposes to consider parameters that directly describe the nanoform (core, surface, or geometry), those that provide an indirect description (other properties and preparation conditions) and descriptors focused not on the nanoform but on the endpoint measurement.

The variety of descriptors reflects how, in nano-QSAR models, the identification of a NM as a particular data point is based on a combination of chemical and physical structures, which could require using experimental parameters. This differs from common QSAR models with molecular substances, where only the chemical structure is used to identify the substance (which usually can be expressed using the SMILES representation). From there, a series of calculated molecular descriptors are obtained that correspond to a single data point determined by the SMILES. However, this is not possible in most nano-QSAR models, which often utilize experimental descriptors such as size and shape to define a specific NM and to model its properties. In this case, those descriptors relate the data point to a particular nanoform with specific properties. This distinction highlights the unique role that experimental descriptors can play in nano-QSAR models. Experimental values in nano-QSAR models are often not derivable from the composition, but rather from “identifying descriptors”, that is, fundamental experimental features that are necessary for the model and that identify a nanoform. For example, the size of a nanoparticle is often used as an “identifying descriptor” because it is a key parameter that determines the properties of the material. However, most of the electronic experimental values obtained from bulk materials discussed above are “derived descriptors”, which are similar to the calculated values, as they are potentially derivable from other features or identifiers such as the SMILES.

Other experimental measures, such as zeta potential, may be used as identifying features and can be considered derived from nanostructural information (as the value will largely depend on the composition). In any case, we consider as “identifying” those features that are required as input data for a prediction and are necessary to make accurate predictions, regardless of whether they are physically bound or not.

According to our analysis, despite the existence of a broader range of options and the need to incorporate structural information, composition-based descriptors remain the norm in nano-QSAR. In spite of the chemical complexity inherent to any extended system (such as a crystal or polymer), most descriptors are simpler than those found for organic molecules, focusing on simplified structural formulas or single elements. In most cases, the composition of a NM is simplified to its major component, ignoring impurities, mixtures, and ligands; when those are incorporated, their proportion is commonly ignored.

Particle size, commonly the measured or nominal value of the diameter, is among the most common features in nano-QSAR models. However, as discussed above, its statistical significance in the predictivity of models is not consistent. This ambiguity might stem from the fundamental shift in properties when transitioning from bulk materials to nanoparticles, making it a quantum leap in terms of behavior. While having a nanoscale size is crucial for exhibiting distinct properties, a specific size within a suitable range might have a less pronounced impact. Consequently, and also because of the limited size variations present in the databases used to train QSAR models, size is often perceived as a parameter of lesser relevance.

In summary, our review discusses and classifies a wide variety of descriptors used for NM predictive modelling. Our analysis highlights the significant efforts made to combine the chemical and structural complexity of the NMs with the objective to obtain convenient descriptors. Our analysis provides a couple of trends that could guide future steps in this field, that is, to calculate descriptors using simplified chemical models and to use experimental properties or conditions as descriptors. Most calculated descriptors are restricted to one component of the core and/or ligands (even assuming part of its chemical composition) and do not include nanostructural information. In contrast, the use of experimental information captures insights on the real structure, but unveils another challenge of the nano-QSAR models, the lack of consistence among the methods and parameters used to characterize and evaluate NMs. In consequence, our proposal classifies the descriptors (mainly calculated) according to the part of the particle that they describe (i.e., the core or the surface ligands) and also discerns among the descriptors used to encode the nanostructural information (mainly experimental) from other experimental data used to obtain an overall description of the NMs, that is, from other properties or the experimental conditions.

## Supporting Information

File 1 All models evaluated in the review, as well as the classification of selected descriptors according to the proposed categories.

## Data Availability

Data sharing is not applicable as no new data was generated or analyzed in this study.
