# Supplementary material for: A review on the structural characterization of nanomaterials for nano-QSAR models
Source: Beilstein J Nanotechnol. 2024 Jul 11;15:854–66. doi: 10.3762/bjnano.15.71 (PMC11250003; doi:10.3762/bjnano.15.71)
Supplement: File 1 — All models evaluated in the review, as well as the classification of selected descriptors according to the proposed categories. [file Beilstein_J_Nanotechnol-15-854-s001.pdf]

BEILSTEIN JOURNAL OF NANOTECHNOLOGY

Supporting Information

for

A review on the structural characterization of nanomaterials for nano-QSAR models

Salvador Moncho, Eva Serrano-Candelas, Jesús Vicente de Julián-Ortiz and Rafael Gozalbes

Beilstein J. Nanotechnol. **2024,** *15,* 854–866. doi:10.3762/bjnano.15.71

All models evaluated in the review, as well as the classification of selected descriptors according to the proposed categories

License and Terms: This is a supporting information file under the terms of the Creative Commons Attribution License (https://creativecommons.org/licenses/by/4.0). Please note that the reuse, redistribution and reproduction in particular requires that the author(s) and source are credited and that individual graphics may be subject to special legal provisions.
The license is subject to the *Beilstein Journal of Nanotechnology* terms and conditions: (https://www.beilstein-journals.org/bjnano/terms)

List of contents:

Table S1: List of models evaluated in the review, as well as the classification of selected descriptors according to the classification presented in this paper.

Table S1: List of models evaluated in the review, as well as the classification of selected descriptors according to the classification presented in this paper. Abbreviatures used in the table: CN: coordination numbers; CNT: carbon nanotubes; Ei: energy of i (CB, conduction band; form, formation, VB, valence band): $El_{(w)}$: descriptors calculated or from literature relating to pure elements (w if weighted for compounds); EC: descriptors based in the electron configuration; EN: descriptors related with electronegativity; Ful.: fullerenes; IS: Ionic strength in the reaction media; LDM: liquid drop model descriptors; Molec.: common molecular descriptors obtained from the SMILES for organic molecules; M_i: descriptor referring only to the metal atom; MOx: metal oxides (element among parenthesis if there is a single composition); NOM: Amount of organic matter in the test media; ON: oxidation number of the metal; Pr_i: binary descriptor indicating the presence/absence of a component or experimental condition; PT: position in the periodic table, QDs: quantum dots; QM: descriptors calculated using electronic methods; S_i: size of the nanomaterial described by its diameter (a subindex can be included referring to different methods or media); SA: surface area; SP: sedimentation potential (calculated); SPR: signaling-pathway responses; Target: the target cell is included as a descriptors (directly or by a perturbation method); R1 and R2: magnetic relaxitivities; ZP: zeta potential; $\#_i$: count of atoms of element/type i.

				Classification					
	**Reference**	**Endpoint**	**NM type**	**A**	**B**	**C**	**D**	**E**	**F**
[1]	Hu, 2009	LD_{50} (cytotoxicity in *E. coli*)	MOx	M_{charge}					
[2]	Fourches, 2010	Cellular uptake in PaCa2 human cells	MOx (Fe)		Molec.				
		Toxicity vector in human cells (4 types)	Mixed			S			
[3]	Sayes, 2010	Membrane damage in human lung cells (LDH test)	MOx			S_{TEM}, S_{Water}, S_{buff}	ZP, R1, R2		
[4]	Burello, 2011b	Oxidative stress potential	MOx	M_{ON}					
[5]	Liu, 2011	Cytotoxicity in BEAS-2	MOx	$\#_{M,O}$, M_{mass}, PT					

	Reference	Endpoint	NM type	Classification					
				A	B	C	D	E	F
[6]	Puzyn, 2011	pEC_{50} (cytotoxicity in *E. coli*)	MOx	QM					
[7]	Rallo, 2011	Luciferase in murine RAW cells	M/Mox				SPR		
[8]	Chau, 2012	Cellular uptake in PaCa2 human cells	MOx (Fe)		Molec				
[9]	Epa, 2012	Smooth cell apoptosis	Mixed	Pr_{Fe2O3}	$Pr_{Dextran}$, Acidity				
		Cellular uptake (HUVEC/ PaCa2)	MOx (Fe)		Molec.				
[10]	Patel, 2012	Cytotoxicity in RAW and BEAS-2B cells	Mixed						
[11]	Toropov, 2012	pEC_{50} (cytotoxicity in *E. coli*)	MOx						
[12]	Ahmed, 2013	pEC_{50} (HIV-protease binding)	Ful.	QM	Molec.				
[13]	Liu, 2013a	Cytotoxicity	MOx			S	ZP, R1, R2		
[14]	Liu, 2013b	EZ metric (Zebrafish embryo)	Mixed					precursors	
[15]	Liu, 2013c	Cytotoxicity in BEASE/RAW (MTS)	MOx						
[16]	Kar, 2014	pEC_{50} (cytotoxicity in *E. coli*)	MOx	EN, M_{charge}					

	Reference	Endpoint	NM type	Classification A	B	C	D	E	F
[17]	Kleandrova, 2014	Multiple: ecotoxicity and toxicity	M/MOx	$EI_{(w)}$					
[18]	Liu, 2014	10 SPRs and 4 cytotoxicity indicators in RAW/BEAS-2B	M/Mox						
[19]	Pathakoti, 2014	pEC_{50} (cytotoxicity in *E. coli*)	MOx	QM		S_{TEM}, S_{hydro}, SA, Mass, V	ZP_{water}, ZP_{media}		Pr_{Light}
[20]	Singh, 2014	several	Mixed						
[21]	Sizochenko, 2014	pEC_{50} (cytotoxicity in *E. coli* and HaCaT)	MOx			LDM			
[22]	Toropov, 2014	Mutagenicity (Ames test in TA100)	Ful.						Pr_{S9}, $Pr_{Irradiation}$, Dose
[23]	Venigalla, 2014	pEC_{50} (cytotoxicity in *E. coli*)	MOx						
[24]	Gajewicz, 2015	HaCaT	MOx						
[25]	Kaweeteerawat, 2015	pEC_{50} (cytotoxicity in *E. coli*)	MOx						
[26]	Mikolajczyk, 2015	Zeta Potential	MOx						

	Reference	Endpoint	NM type	Classification A	B	C	D	E	F
[27]	Sizochenko, 2015	Cytotoxicity in BEASE/RAW (MTS)	MOx						
[28]	Toropov, 2015b	Mutagenicity (Ames test in TA100)	Ful./CNT				Id		Pr_{S9}, $Pr_{incub.}$, Dose
[29]	Toropov , 2015a	Mutagenicity (Ames test in TA100)	CNT						Pr_{S9}, $Pr_{incub.}$, Dose
[30]	Toropova, 2015	Cell viability (HK-2/LLC-PK1)	SiO_2		$\#_M$		S_{bin}		Dose, time
[31]	Cassano, 2016	pEC_{25} (WST-1) Several	SiO_2				S_{bin}, R_{bin}	ZP_{bin}	Time, CL,
[32]	Jagiello, 2016	HIV-protease binding	Ful.						
[33]	Kar, 2016	pEC_{50} (cytotoxicity in *E. coli* and HaCaT)	MOx						
[34]	Le, 2016	Cell viability/LDH	MOx (Zn)						
[35]	Manganelli, 2016	Cell viability in HEK cells	SiO_2			S			Dose, Time
[36]	Mu, 2016	E.Coli	MOx	EN, $M_{softness}$, M_{IP}					

Reference	Endpoint	NM type	Classification A	B	C	D	E	F
[37] Pan, 2016	E. Coli / HaCaT	MOx						
[38] Toropova, 2016a	Mutagenicity (Ames test in TA100 and E. coli)	Ful.						Pr_{S9}, Pr_{irrad}, Dose
[39] Toropova, 2016b	Mutagenicity (Ames test in TA100)	CNT				Id		Pr_{S9},, Dose
[40] Toropova, 2016c	pEC_{50} (cytotoxicity in *E. coli* and HaCaT)	MOx	SMILES					Target
[41] Basant, 2017	pEC_{50} (cytotoxicity in *E. coli* and HaCaT)	MOx	several					Pr_{Light}, Target
[42] Concu, 2017	Toxicity (general)	M/Mox	El_{Volume}, EN, $El_{Polarizability}$		S, shape		EC_{50}_oth	time
[43] Fjodorova, 2017	pEC_{50} (cytotoxicity to *E. coli)*	MOx	$\#_{M,O}$, EN, PT, M_{charge}					
[44] Manganelli, 2017	Cell viability	SiO_2			S_{bin}			Dose, Time
[45] Sizochenko, 2017	Zebrafish Hatching Enzyme ZHE1	MOx						
[46] Zhou, 2017	pEC_{50} (cytotoxicity to *E. coli)*	MOx						

Reference	Endpoint	NM type	Classification A	B	C	D	E	F
[47] Choi, 2018	Cell viability several	MOx	E_{CB}, E_{VB}, ΔH_f		S_{TEM}, S_{DLS}			
[48] De, 2018	pEC_{50} (cytotoxicity to *E. coli*/HaCaT) and % EI (Zebrafish)	MOx	M_{ON}, EN, Valence			Tox_{other}		
[49] Mikolajczyk, 2018	pEC50 (cytotoxicity to CHO-K1)	MOx (Ti)	M_{IP}, M_{EA}, M_{hard}, M_{ads}					
[50] Shin, 2018	Immobilization *D. magna*	several	El, Several	Coating	S_{TEM}, S_{agg}, Shape	ZP		$Pr_{Exp. cond}$ Time, IS, NOM, Number DM,
[51] Sizochenko, 2018	pEC50 (several targets)	MOx	M_{ch}, El_{ion_rad}, EN, E_{gap}		$R_{Wigner-Seitz}$			
[52] Trinh, 2018		Ful.						
[52] Buglak, 2019	cell viability several	CNT			S, Lenght, Area			Dose,Time ,CL, Method
[53] Cai, 2019	EC50 (*D. Magna*)	MOx (Ti)						

			Classification					
**Reference**	**Endpoint**	**NM type**	**A**	**B**	**C**	**D**	**E**	**F**
[54] Choi, 2019	% (cell viability in HaCaT and BEAS)	MOx/Ag	$\#_{elements}$		S_{TEM}, S_{DLS}	Surface charge		Assay, dose
[55] Kuz'min, 2019	E.Coli/Hacat	MOx	several		LDM, S			
[56] Mikolajczyk, 2019	pEC_{50} (cytoxocity on CHO-K1)	MOx (Ti)		EN				
	Photodegradation of phenol	MOx (Ti)		$\%_{Pd}$, SP	SA_{BET}			
[57] Ojha, 2019	Cellular uptake in PaCa2/HUVEC/U937	MOx (Fe)						
[58] Ambure, 2020	Cytoxicity/Genotoxicity	MOx		Molec				
[59] Cao, 2020	Cytoxicity A549 cells	MOx	$M_{charge,}$ $M_{\%w}$					
[60] Halder, 2020	Genotoxicity	MOx	$El_{(w)}$					
[61] Huang, 2020	Inflammatory potential IL1beta production	MOx						
[62] Kotzabasaki, 2020	Cell viability	FeOx						
[63] Kudrinskiy, 2020	MIC (S. cerevisae)	Ag			S, SA	ZP, Cmax, kPO		

Reference	Endpoint	NM type	Classification A	B	C	D	E	F
[64] Papadiamantis, 2020	Cell viability	MOx	Crystal, CN		S, SA, TSA, Shydro,	ZP, PZZP,		Assay
[65] RybińskaFryca, 2020	Photodegradation of phenol	MOx (Ti)		$\#_{N,C}$	SA_{BET}		Precursor (IL, cation, anion)	
	pEC_{50} (cytotoxicity to *E. coli)*	MOx (Ti)		EN, $M_{\%mol}$				
	Zeta potential (in media)		HOMO/$\#_M$		S_{TEM}	ZP_{water}		
[66] Gul, 2021	cell viability	Several					Synthesys method	
[67] Jung, 2021	pLC_{50} (fish)	Ag			S	ZP, $pLC_{50Daphn}$		
[68] Kumar, 2021	cell viability	QDs						
[69] Qi, 2021	pEC50 (cytotoxicity to CHO-K1)	MOx (Ti)		$M_{mol\_fraction}$				
[70] Sifonte, 2021	pEC_{50} HaCaT	MOx						
[71] Toropova, 2021	Cell viability	MOx	Formula, H_{form}, Ecb, Evb, EN.		S_{TEM}, S_{hydro}			CL, Species, Origin, Cell-type, Time,

Reference	Endpoint	NM type	A	B	C	D	E	F
					Classification			
								Dose, Assay
[72] Varsou, 2021	Immobilization of *D. Magna*	Ag/MOx (Ti)			S_{TEM}, S_{DLS},	ZP, Cond. El Mob.	Aging	
[73] Yuan, 2021	Cell proliferation rate HK2	M+MOx (Ti)						
[74] Zhang, 2021	Interaction energy (calculated) with SARS-CoV-2 proteins	Ful./CNT/ Graph.			D, L, SA, Angle			
[75] Shin, 2021	Cell viability in human lung cells	MOx (Ti)	EC	EC				
[76] Banerjee, 2023	pEC_{50} (cytotoxicity to CHO-K1)	MOx (Ti)	EN, EA, ΔH_f		SA			
[77] Na, 2023	$EC_{10/50}$ (toxicity to *A. fischeri*)	MOx			S_{NM}, S_{DLS}, SA	ZP		

References

1. Hu, X.; Cook, S.; Wang, P.; Hwang, H. min *Science of the Total Environment* **2009**, *407*, 3070–3072, doi:10.1016/j.scitotenv.2009.01.033
2. Fourches, D.; Pu, D.; Tassa, C.; Weissleder, R.; Shaw, S.Y.; Mumper, R.J.; Tropsha, A. *ACS Nano* **2010**, *4*, 5703–5712, doi:10.1021/nn1013484
3. Sayes, C.; Ivanov, I. , doi:10.1111/j.1539-6924.2010.01438.x
4. Burello, E.; Worth, A.P. *Nanotoxicology* **2011**, *5*, 228–235, doi:10.3109/17435390.2010.502980
5. Liu, R.; Rallo, R.; George, S.; Ji, Z.; Nair, S.; Nel, A.E.; Cohen, Y. *Small* **2011**, *7*, doi:10.1002/smll.201002366
6. Puzyn, T.; Rasulev, B.; Gajewicz, A.; Hu, X.; Dasari, T.P.; Michalkova, A.; Hwang, H.M.; Toropov, A.; Leszczynska, D.; Leszczynski, J. *Nat Nanotechnol* **2011**, *6*, 175–178, doi:10.1038/nnano.2011.10
7. Rallo, R.; France, B.; Liu, R.; Nair, S.; George, S.; Damoiseaux, R.; Giralt, F.; Nel, A.; Bradley, K.; Cohen, Y. *Environ Sci Technol* **2011**, *45*, 1695–1702, doi:10.1021/ES103606X/SUPPL_FILE/ES103606X_SI_001.PDF
8. Chau, Y.T.; Yap, C.W. *RSC Adv* **2012**, *2*, 8489–8496, doi:10.1039/c2ra21489j
9. Epa, V.C.; Burden, F.R.; Tassa, C.; Weissleder, R.; Shaw, S.; Winkler, D.A. *Nano Lett* **2012**, *12*, 5808–5812, doi:10.1021/nl303144k
10. Patel, T.R. Ph.D. Thesis: Bayesian Methods in the Quantitative Risk Assessment and Toxicity Profiling of Engineered Nanomaterials, University of California Los Angeles, 2012
11. Toropov, A.A.; Toropova, A.P.; Benfenati, E.; Gini, G.; Puzyn, T.; Leszczynska, D.; Leszczynski, J. *Chemosphere* **2012**, *89*, 1098–1102, doi:10.1016/j.chemosphere.2012.05.077
12. Ahmed, L.; Rasulev, B.; Turabekova, M.; Leszczynska, D.; Leszczynski, J. *Org Biomol Chem* **2013**, *11*, doi:10.1039/c3ob40878g
13. Liu, R.; Rallo, R.; Weissleder, R.; Tassa, C.; Shaw, S.; Cohen, Y. *Small* **2013**, *9*, 1842–1852, doi:10.1002/smll.201201903
14. Liu, X.; Tang, K.; Harper, S.; Harper, B.; Steevens, J.A.; Xu, R. *Int J Nanomedicine* **2013**, *8*, 31–43, doi:10.2147/IJN.S40742
15. Liu, R.; Zhang, H.Y.; Ji, Z.X.; Rallo, R.; Xia, T.; Chang, C.H.; Nel, A.; Cohen, Y. *Nanoscale* **2013**, *5*, 5644–5653, doi:10.1039/C3NR01533E
16. Kar, S.; Gajewicz, A.; Puzyn, T.; Roy, K.; Leszczynski, J. *Ecotoxicol Environ Saf* **2014**, *107*, 162–169, doi:10.1016/j.ecoenv.2014.05.026
17. Kleandrova, V. V.; Luan, F.; González-Díaz, H.; Ruso, J.M.; Speck-Planche, A.; Cordeiro, M.N.D.S. *Environ Sci Technol* **2014**, *48*, 14686–14694, doi:10.1021/es503861x
18. Liu, R.; France, B.; George, S.; Rallo, R.; Zhang, H.; Xia, T.; Nel, A.E.; Bradley, K.; Cohen, Y. *Analyst* **2014**, *139*, 943–953, doi:10.1039/C3AN01409F
19. Pathakoti, K.; Huang, M.J.; Watts, J.D.; He, X.; Hwang, H.M. *J Photochem Photobiol B* **2014**, *130*, 234–240, doi:10.1016/j.jphotobiol.2013.11.023

20. Singh, K.P.; Gupta, S. *RSC Advances* **2014**, *4*, 13215–13230
21. Sizochenko, N.; Rasulev, B.; Gajewicz, A.; Kuz'Min, V.; Puzyn, T.; Leszczynski, J. *Nanoscale* **2014**, *6*, doi:10.1039/c4nr03487b
22. Toropov, A.A.; Toropova, A.P. *Chemosphere* **2014**, *104*, 262–264, doi:10.1016/j.chemosphere.2013.10.079
23. Venigalla, S.; Dhail, S.; Ranjan, P.; Jain, S.; Chakraborty, T. *Former: Ann. West Univ. Timisoara-Series Chem* **2014**, *23*, 123–130
24. Gajewicz, A.; Schaeublin, N.; Rasulev, B.; Hussain, S.; Leszczynska, D.; Puzyn, T.; Leszczynski, J. *Nanotoxicology* **2015**, *9*, 313–325, doi:10.3109/17435390.2014.930195
25. Kaweeteerawat, C.; Ivask, A.; Liu, R.; Zhang, H.; Chang, C.H.; Low-Kam, C.; Fischer, H.; Ji, Z.; Pokhrel, S.; Cohen, Y.; et al. *Environ Sci Technol* **2015**, *49*, 1105–1112, doi:10.1021/es504259s
26. Mikolajczyk, A.; Gajewicz, A.; Rasulev, B.; Schaeublin, N.; Maurer-Gardner, E.; Hussain, S.; Leszczynski, J.; Puzyn, T. *Chemistry of Materials* **2015**, *27*, 2400–2407, doi:10.1021/cm504406a
27. Sizochenko, N.; Rasulev, B.; Gajewicz, A.; Mokshyna, E.; Kuz'min, V.E.; Leszczynski, J.; Puzyn, T. *RSC Adv* **2015**, *5*, 77739–77745, doi:10.1039/C5RA11399G
28. Toropov, A.A.; Toropova, A.P. *Chemosphere* **2015**, *139*, 18–22, doi:10.1016/j.chemosphere.2015.05.042
29. Toropov, A.A.; Toropova, A.P. *Chemosphere* **2015**, *124*, 40–46, doi:10.1016/j.chemosphere.2014.10.067
30. Toropova, A.P.; Toropov, A.A.; Benfenati, E. *SAR QSAR Environ Res* **2015**, *26*, 29–40, doi:10.1080/1062936X.2014.984327
31. Cassano, A.; Robinson, R.L.M.; Palczewska, A.; Puzyn, T.; Gajewicz, A.; Tran, L.; Manganelli, S.; Cronin, M.T.D. *ATLA Alternatives to Laboratory Animals* **2016**, *44*, doi:10.1177/026119291604400603
32. Jagiello, K.; Grzonkowska, M.; Swirog, M.; Ahmed, L.; Rasulev, B.; Avramopoulos, A.; Papadopoulos, M.G.; Leszczynski, J.; Puzyn, T. *Journal of Nanoparticle Research* **2016**, *18*, doi:10.1007/s11051-016-3564-1
33. Kar, S.; Gajewicz, A.; Roy, K.; Leszczynski, J.; Puzyn, T. *Ecotoxicol Environ Saf* **2016**, *126*, 238–244, doi:10.1016/j.ecoenv.2015.12.033
34. Le, T.C.; Yin, H.; Chen, R.; Chen, Y.; Zhao, L.; Casey, P.S.; Chen, C.; Winkler, D.A. *Small* **2016**, *12*, 3568–3577, doi:10.1002/SMLL.201600597
35. Manganelli, S.; Leone, C.; Toropov, A.A.; Toropova, A.P.; Benfenati, E. *Chemosphere* **2016**, *144*, 995–1001, doi:10.1016/J.CHEMOSPHERE.2015.09.086
36. Mu, Y.; Wu, F.; Zhao, Q.; Ji, R.; Qie, Y.; Zhou, Y.; Hu, Y.; Pang, C.; Hristozov, D.; Giesy, J.P.; et al. *Nanotoxicology* **2016**, *10*, 1207–1214, doi:10.1080/17435390.2016.1202352
37. Pan, Y.; Li, T.; Cheng, J.; Telesca, D.; Zink, J.I.; Jiang, J. *RSC Adv* **2016**, *6*, 25766–25775, doi:10.1039/C6RA01298A
38. Toropova, A.P.; Toropov, A.A.; Veselinović, A.M.; Veselinović, J.B.; Benfenati, E.; Leszczynska, D.; Leszczynski, J. *Ecotoxicol Environ Saf* **2016**, *124*, 32–36, doi:10.1016/j.ecoenv.2015.09.038
39. Toropova, A.P.; Toropov, A.A.; Rallo, R.; Leszczynska, D.; Leszczynski *Int. J. Environ. Res* **2016**, *10*, 59–64

40. Toropova, A.P.; Toropov, A.A.; Manganelli, S.; Leone, C.; Baderna, D.; Benfenati, E.; Fanelli, R. *NanoImpact* **2016**, *1*, 60–64, doi:10.1016/j.impact.2016.04.003
41. Basant, N.; Gupta, S. *Nanotoxicology* **2017**, *11*, 339–350, doi:10.1080/17435390.2017.1302612
42. Concu, R.; Kleandrova, V. V.; Speck-Planche, A.; Cordeiro, M.N.D.S. *http://dx.doi.org/10.1080/17435390.2017.1379567* **2017**, *11*, 891–906, doi:10.1080/17435390.2017.1379567
43. Fjodorova, N.; Novic, M.; Gajewicz, A.; Rasulev, B. *Nanotoxicology* **2017**, *11*, 475–483, doi:10.1080/17435390.2017.1310949
44. Manganelli, S.; Benfenati, E. *Methods in Molecular Biology* **2017**, *1601*, 275–290, doi:10.1007/978-1-4939-6960-9_22/FIGURES/6
45. Sizochenko, N.; Leszczynska, D.; Leszczynski, J. *Nanomaterials* **2017**, *7*, doi:10.3390/nano7100330
46. Zhou, Z.; Tang, X.; Dai, W.; Shi, J.; Chen, H. *Can J Chem* **2017**, *95*, 863–866, doi:10.1139/cjc-2017-0172
47. Choi, J.S.; Ha, M.K.; Trinh, T.X.; Yoon, T.H.; Byun, H.G. *Sci Rep* **2018**, *8*, doi:10.1038/s41598-018-24483-z
48. De, P.; Kar, S.; Roy, K.; Leszczynski, J. *Environ Sci Nano* **2018**, *5*, doi:10.1039/C8EN00809D
49. Mikolajczyk, A.; Gajewicz, A.; Mulkiewicz, E.; Rasulev, B.; Marchelek, M.; Diak, M.; Hirano, S.; Zaleska-Medynska, A.; Puzyn, T. *Environ Sci Nano* **2018**, *5*, doi:10.1039/c8en00085a
50. Shin, H.K.; Seo, M.; Shin, S.E.; Kim, K.Y.; Park, J.W.; No, K.T. *Environ Sci Nano* **2018**, *5*, 765–775, doi:10.1039/c7en01127j
51. Sizochenko, N.; Mikolajczyk, A.; Jagiello, K.; Puzyn, T.; Leszczynski, J.; Rasulev, B. *Nanoscale* **2018**, *10*, 582–591, doi:10.1039/C7NR05618D
52. Trinh, T.X.; Choi, J.S.; Jeon, H.; Byun, H.G.; Yoon, T.H.; Kim, J. *Chem Res Toxicol* **2018**, *31*, 183–190, doi:10.1021/acs.chemrestox.7b00303
53. Cai, Y.; Nowack, B.; Wigger, H. *NanoImpact* **2019**, *15*, doi:10.1016/j.impact.2019.100180
54. Choi, J.S.; Trinh, T.X.; Yoon, T.H.; Kim, J.; Byun, H.G. *Chemosphere* **2019**, *217*, 243–249, doi:10.1016/j.chemosphere.2018.11.014
55. Kuz'min, V.E.; Ognichenko, L.N.; Sizochenko, N.; Chapkin, V.A.; Stelmakh, S.I.; Shyrykalova, A.O.; Leszczynski, J. *International Journal of Quantitative Structure-Property Relationships* **2018**, *4*, 28–40, doi:10.4018/ijqspr.2019010103
56. Mikolajczyk, A.; Sizochenko, N.; Mulkiewicz, E.; Malankowska, A.; Rasulev, B.; Puzyn, T. *Nanoscale* **2019**, *11*, 11808–11818, doi:10.1039/C9NR01162E
57. Ojha, P.K.; Kar, S.; Roy, K.; Leszczynski, J. *Nanotoxicology* **2019**, *13*, 14–34, doi:10.1080/17435390.2018.1529836
58. Ambure, P.; Ballesteros, A.; Huertas, F.; Camilleri, P.; Barigye, S.J.; Gozalbes, R. *International Journal of Quantitative Structure-Property Relationships* **2020**, *5*, 83–100, doi:10.4018/ijqspr.20201001.oa2
59. Cao, J.; Pan, Y.; Jiang, Y.; Qi, R.; Yuan, B.; Jia, Z.; Jiang, J.; Wang, Q. *Green Chemistry* **2020**, *22*, 3512–3521, doi:10.1039/d0gc00933d
60. Halder, A.K.; Melo, A.; Cordeiro, M.N.D.S. *Chemosphere* **2020**, *244*, doi:10.1016/j.chemosphere.2019.125489

61. Huang, Y.; Li, X.; Xu, S.; Zheng, H.; Zhang, L.; Chen, J.; Hong, H.; Kusko, R.; Li, R. *Environ Health Perspect* **2020**, *128*, 1–13, doi:10.1289/EHP6508
62. Kotzabasaki, M.I.; Sotiropoulos, I.; Sarimveis, H. *RSC Adv* **2020**, *10*, 5385–5391, doi:10.1039/c9ra09475j
63. Kudrinskiy, A.; Zherebin, P.; Gusev, A.; Shapoval, O.; Pyee, J.; Lisichkin, G.; Krutyakov, Y. *Nanomaterials* **2020**, *10*, 1–22, doi:10.3390/nano10081459
64. Papadiamantis, A.G.; Jänes, J.; Voyiatzis, E.; Sikk, L.; Burk, J.; Burk, P.; Tsoumanis, A.; Ha, M.K.; Yoon, T.H.; Valsami-Jones, E.; et al. *Nanomaterials* **2020**, *10*, 1–19, doi:10.3390/nano10102017
65. Rybińska-Fryca, A.; Mikolajczyk, A.; Puzyn, T. *Nanoscale* **2020**, *12*, 20669–20676, doi:10.1039/d0nr05220e
66. Gul, G.; Yildirim, R.; Ileri-Ercan, N. *Environ Sci Nano* **2021**, *8*, 937–949, doi:10.1039/D0EN01240H
67. Jung, U.; Lee, B.; Kim, G.; Shin, H.K.; Kim, K.T. *Chemosphere* **2021**, *283*, doi:10.1016/j.chemosphere.2021.131164
68. Kumar, A.; Kumar, P. *J Hazard Mater* **2021**, *402*, 123777, doi:10.1016/J.JHAZMAT.2020.123777
69. Qi, R.; Pan, Y.; Cao, J.; Yuan, B.; Wang, Y.; Jiang, J. *Environ Sci Nano* **2021**, *8*, 927–936, doi:10.1039/D0EN01266A
70. Peláez Sifonte, E.; Antonio Castro-Smirnov, F.; Adrian Soutelo Jimenez, A.; Raúl González Diez, H.; Guzmán Martínez, F. *Journal of Nanoparticle research* **2021**, *23*, 161, doi:10.1007/s11051-021-05288-0
71. Toropova, A.P.; Toropov, A.A.; Leszczynski, J.; Sizochenko, N. *Environ Toxicol Pharmacol* **2021**, *86*, doi:10.1016/j.etap.2021.103665
72. Varsou, D.D.; Ellis, L.J.A.; Afantitis, A.; Melagraki, G.; Lynch, I. *Chemosphere* **2021**, *285*, doi:10.1016/j.chemosphere.2021.131452
73. Yuan, B.; Wang, P.; Sang, L.; Gong, J.; Pan, Y.; Hu, Y. *Ecotoxicol Environ Saf* **2021**, *208*, doi:10.1016/j.ecoenv.2020.111634
74. Zhang, F.; Wang, Z.; Vijver, M.G.; Peijnenburg, W.J.G.M. *Ecotoxicol Environ Saf* **2021**, *219*, doi:10.1016/j.ecoenv.2021.112357
75. Shin, H.K.; Kim, S.; Yoon, S. *NanoImpact* **2021**, *21*, 100298, doi:10.1016/J.IMPACT.2021.100298
76. Banerjee, A.; Kar, S.; Pore, S.; Roy, K. *https://doi.org/10.1080/17435390.2023.2186280* **2023**, *17*, 78–93, doi:10.1080/17435390.2023.2186280
77. Na, M.; Nam, S.H.; Moon, K.; Kim, J. *Environ Sci Nano* **2023**, *10*, 325–337, doi:10.1039/D2EN00672C
